# A Bidirectional C1q/TNF-Related Protein Signature Is Associated with Disease Activity in Behçet’s Disease: A Cross-Sectional Study

**DOI:** 10.3390/diagnostics16183035

**Published:** 2026-09-19

**Authors:** Ahmet Karataş, Ramazan Fazıl Akkoç, Burak Öz

**Affiliations:** 1 Division of Rheumatology, Department of Internal Medicine, Faculty of Medicine, Fırat University, 23119 Elazığ, Türkiye; 2 Department of Anatomy, Faculty of Medicine, Kütahya Health Sciences University, 43100 Kütahya, Türkiye

**Keywords:** Behçet’s disease, disease activity, C1q/TNF-related protein, CTRP12, CTRP6, adipokine, biomarker, vasculitis

## Abstract

**Background/Objectives**: Distinguishing active from quiescent Behçet’s disease (BD) is difficult because conventional acute-phase reactants perform inconsistently. C1q/TNF-related proteins (CTRPs) are adiponectin paralogues; several members have been reported to exert opposing immunometabolic actions in experimental systems, although the evidence is uneven across the family and no member has been examined in BD. **Methods**: In this cross-sectional study, eight CTRPs (CTRP1, CTRP4, CTRP6, CTRP9, CTRP12, CTRP13, CTRP14, CTRP15) were measured in serum using enzyme-linked immunosorbent assay in 30 patients with active BD, 30 with inactive BD and 30 healthy controls. Disease activity was defined by a Behçet’s Disease Current Activity Form (BDCAF) score of ≥2. False discovery rate correction was applied separately to the eight omnibus and eight active-versus-inactive biomarker comparisons. Logistic models were adjusted for age, sex, body mass index and disease duration; a composite index combined the analytes changing in opposite directions. **Results**: Six analytes differed between active and inactive disease after correction (q = 0.004–0.041): CTRP1, CTRP4, CTRP6 and CTRP14 were higher and CTRP12 and CTRP15 lower in active disease, with five correlating with the BDCAF score in the same directions. Four showed nominally significant associations in the adjusted models (CTRP12, CTRP14, CTRP6 and CTRP15), whereas CTRP1 and CTRP4 did not; adjusted odds ratios per doubling ranged from 0.27 (CTRP12, 95% CI 0.11–0.71) to 2.73 (CTRP14, 1.16–6.44). The composite index achieved an area under the curve of 0.866 (95% CI 0.765–0.944); C-reactive protein alone reached 0.810 (0.682–0.915) and the two combined 0.916 (0.839–0.974). Bootstrap internal validation, in which the selection of components was repeated in each resample, gave an out-of-bag area under the curve of 0.812 (0.649–0.946). The index remained associated with activity after adjustment for C-reactive protein (*p* = 0.002); adding the index to C-reactive protein did not significantly increase discrimination (ΔAUC +0.106, 95% CI −0.002 to +0.214; DeLong *p* = 0.055). **Conclusions**: In this cross-sectional study, active BD was associated with a coordinated bidirectional CTRP pattern that paralleled clinical activity. The composite index remained associated with activity after adjustment for C-reactive protein, but its incremental discrimination over C-reactive protein did not reach statistical significance; longitudinal and external validation are required.

## 1. Introduction

Behçet’s disease is a chronic, relapsing systemic vasculitis characterised by recurrent oral and genital ulceration, ocular inflammation, skin lesions and variable vascular, articular and neurological involvement [1,2]. Its pathogenesis combines autoinflammatory and adaptive features, with neutrophil hyperactivity, T helper 17 skewing and endothelial dysfunction all implicated [3]. Because the disease alternates between flares and quiescence, and immunosuppressive escalation is guided largely by clinical judgement [4], an objective measure of activity would be of direct practical value. Composite clinical instruments such as the Behçet’s Disease Current Activity Form quantify the burden of manifestations [5]. No circulating marker, however, reliably tracks activity. C-reactive protein and the erythrocyte sedimentation rate are often normal during clinically evident flares, particularly when activity is confined to mucocutaneous surfaces.

Adipose tissue is an immunologically active organ. The C1q/tumour necrosis factor-related proteins form a conserved family of fifteen secreted glycoproteins that share the globular C1q domain of adiponectin and regulate glucose and lipid handling, vascular tone and inflammation [6,7,8]. Members of this family are not functionally interchangeable: CTRP1 and CTRP6 exert predominantly pro-inflammatory actions, whereas CTRP3, CTRP9, CTRP12, CTRP13 and CTRP15 behave protectively in models of atherosclerosis, renal injury and colitis [6,8,9]. This classification rests largely on cardiometabolic models and does not extend to every member, and for some members, the experimental and clinical directions do not coincide. This divergence is nevertheless why a panel approach is informative, since examining a single member risks capturing only one arm of a bidirectional process.

Adiponectin dysregulation is well documented in Behçet’s disease. A meta-analysis of fourteen studies found resistin and adiponectin elevated and visfatin reduced, but only after adjustment for age, sex and body mass index, illustrating how sensitive these comparisons are to demographic confounding [10]. Leptin is elevated [11], as is resistin once demographic factors are taken into account [10], whereas visfatin and omentin are reduced [10,12,13]. Elevated adiponectin has been reported to predispose to the disease [14], and asprosin was recently shown to rise with disease activity and organ involvement [15]. A broader immunometabolic panel found soluble tumour necrosis factor receptors and leptin elevated even in quiescent disease [16]. These observations indicate that the balance between pro-inflammatory and protective adipose-derived mediators is displaced in Behçet’s disease. No study has yet examined the CTRP family, whose members span both sides of that balance.

Mechanistic work supports a role for several members in pathways relevant to this disease. CTRP1 expression in adipose tissue is induced by tumour necrosis factor alpha and interleukin-1 beta [17], and it links macrophage lipid handling to vascular inflammation [18]. It aggravates cardiac fibrosis through NOX2/p38 signalling [19] and post-infarction inflammation through Toll-like receptor 4 [20]. Its actions are nonetheless context-dependent, with circulating and tissue concentrations diverging in chronic kidney disease [21] and with protective metabolic effects in other settings [22]. CTRP6 links adipose inflammation to insulin resistance [23], promotes the macrophage inflammatory response [24] and restrains anti-inflammatory, M2-like macrophage polarisation [25], yet also acts as an endogenous complement regulator capable of treating induced arthritis [26]. On the protective side, CTRP12 is inversely associated with coronary artery disease [27,28] and with inflammatory markers in chronic obstructive pulmonary disease [29]; it limits inflammasome activation [30] and promotes M2-like polarisation [31]. CTRP15, although grouped with the protective members on experimental grounds [9], has been reported at higher rather than lower concentrations in coronary artery disease [32], so its clinical direction is not settled. CTRP14 has almost no immunological characterisation: targeted deletion indicates that it is largely dispensable for metabolic homeostasis [33] and atherogenesis [34], and the only clinical biomarker report concerns Alzheimer’s disease [35]. Clinical data on the family in immune-mediated inflammatory disease remain sparse. They comprise CTRP5 in rheumatoid arthritis [36], CTRP6 in systemic lupus erythematosus [37] and a negative report in multiple sclerosis [38].

A circulating marker can be asked to do three different things in this setting. It may contribute to the diagnosis of BD; it may reflect current disease activity in patients already diagnosed; or its concentration may carry a mechanistic implication. The present study addresses the second point. Our primary objective was to test whether circulating CTRP concentrations differ between patients with active and inactive Behçet’s disease, and whether a multi-marker score derived from analytes changing in opposite directions is associated with clinical activity. C-reactive protein was measured alongside the panel, but not as a comparator that the CTRP score would be expected to outperform. CTRPs are adipose-derived immunometabolic mediators rather than hepatic acute-phase proteins, so comparing the two establishes whether any CTRP signal is independent of the conventional acute-phase response. Healthy controls were included to provide a reference range rather than to evaluate diagnostic performance. We hypothesised that, if the CTRP family participates in the inflammatory activity of the disease, members with pro-inflammatory and protective actions would move in opposite directions during clinically active disease.

## 2. Materials and Methods

### 2.1. Study Design and Participants

This cross-sectional study was conducted at the Division of Rheumatology, Faculty of Medicine, Fırat University. Adult patients with Behçet’s disease fulfilling the International Study Group criteria [39] were recruited prospectively from among those followed in the Division of Rheumatology, during their scheduled visits, between January 2025 and January 2026. Recruitment was quota-based rather than consecutive: patients judged clinically to have active or inactive disease were enrolled alternately, and enrolment in each stratum stopped once thirty patients had been reached. This produced the equal group sizes reported here, but it also means that the study population is not a consecutive series and does not represent the full activity spectrum seen in the clinic. Patients attend at regular intervals for assessment of activity, screening for organ involvement and adjustment of immunosuppressive therapy. All were previously diagnosed patients under active follow-up rather than newly referred cases, and eligible attenders were invited as they presented until each stratum was complete. Disease activity was assessed on the day of blood sampling by a rheumatologist using the Behçet’s Disease Current Activity Form (BDCAF) [5]. The form records twelve clinical manifestations present during the preceding four weeks, each scored as present or absent, giving a total of 0–12. For the present study, a BDCAF score of ≥2 defined active disease and a score of <2 defined inactive disease. The observed distribution was distinctly bimodal: every patient classified as inactive scored 0 or 1 and every patient classified as active scored 6 or above, and no patient scored between 2 and 5. This gap follows from the recruitment design rather than from chance. Patients were enrolled into one of two clinically unambiguous strata, so those with intermediate activity were not recruited into either. The classification is therefore insensitive to the exact BDCAF cut-off, but the two groups represent the ends of the activity range rather than a continuum. The rheumatologist performing the activity assessment was unaware of any biomarker result, all of which were generated after clinical classification had been completed. Thirty patients with active disease and thirty with inactive disease were included. Thirty healthy volunteers were recruited over the same period from hospital staff and from relatives accompanying patients at unrelated outpatient clinics. They were required to have no known inflammatory, rheumatic, malignant or metabolic disease, no regular medication and normal routine laboratory results. They were recruited prospectively over the same period specifically for this study rather than drawn from any pre-existing cohort or biobank. Recruitment was directed towards the age range and sex distribution of the patient groups so that the comparison would not be confounded by demographic imbalance. Selection was otherwise unrestricted among those who volunteered, and no screening on any laboratory value beyond the routine tests listed above was applied. The similarity between groups reflects this deliberate frequency matching rather than selection on any characteristic related to the analytes under study. No formal a priori power calculation was performed. The sample size was fixed in advance at thirty patients per stratum, and recruitment stopped once that number was reached in each. Exclusion criteria were pregnancy, active infection, diabetes mellitus, known cardiac failure, chronic kidney disease and malignancy. Demographic characteristics, disease duration, organ involvement and treatment received were recorded from clinical records. Treatment doses and duration were not systematically recorded. Organ involvement denotes the cumulative phenotype: any ocular, vascular, articular or neurological manifestation documented at any point in the disease course. It is not the same as involvement that was clinically active when blood was drawn. Recurrent oral ulceration is obligatory under the International Study Group criteria, so all 60 patients had mucocutaneous involvement by definition. A patient with previous deep vein thrombosis or uveitis, now quiescent, is counted in the phenotype but scores zero on the corresponding BDCAF item, since that form records only what has been present in the preceding four weeks. The study protocol was approved by the Non-Interventional Research Ethics Committee of Fırat University (session number 2024/15-34, 19 December 2024) and all participants gave written informed consent.

### 2.2. Laboratory Measurements

Venous blood was drawn after an overnight fast. Routine haematological and biochemical parameters, erythrocyte sedimentation rate and C-reactive protein were measured on the day of sampling. Serum for biomarker analysis was separated by centrifugation and stored at −20 °C until analysis. All analytes were measured using a sandwich enzyme-linked immunosorbent assay according to the manufacturer’s instructions. Kits came from a single supplier (YL Biont, Shanghai YL Biotech Co., Ltd., Shanghai, China): CTRP1 (catalogue number YLA4669HU; assay range 0.05–30 ng/mL, sensitivity 0.02 ng/mL), CTRP4 (YLA4671HU; 0.05–30 ng/mL, 0.03 ng/mL), CTRP6 (YLA4672HU; 0.05–15 ng/mL, 0.019 ng/mL), CTRP9 (YLA4673HU; 0.3–90 ng/mL, 0.13 ng/mL), CTRP12 (YLA1121HU; 5–2000 pg/mL, 2.53 pg/mL), CTRP13 (YLA4727HU; 10–3000 pg/mL, 4.67 pg/mL), CTRP14 (YLA4675HU; 0.5–200 ng/mL, 0.25 ng/mL) and CTRP15 (YLA4253HU; 2–600 pg/mL, 1.02 pg/mL). Ranges and sensitivities are those stated in the kit inserts. Every measured concentration fell inside the corresponding range, so no value had to be extrapolated below the lowest standard or censored at the top of the curve, and no imputation for out-of-range results was required. For every analyte, all 90 samples were assayed alongside the standard curve on a single microplate from a single reagent lot, so no sample was measured on a second plate and between-plate variation could not arise. Samples were not diluted and, because of the limited serum volume available, were assayed once rather than in duplicate. The investigator performing the assays was blinded to group allocation. The manufacturer reports intra-assay and inter-assay coefficients of variation below 8% and 10% respectively for all kits; these figures are taken from the kit inserts and were not independently verified in our laboratory. Because samples were not measured in duplicate, within-study analytical coefficients of variation could not be calculated. Randomization of sample order was not documented in the study records.

### 2.3. Statistical Analysis

Distributional assumptions were assessed with the Shapiro–Wilk test. Because most analytes deviated from normality, continuous variables are presented as medians with interquartile ranges. These were compared across the three groups with the Kruskal–Wallis test, followed by pairwise Mann–Whitney U tests. Categorical variables were compared with the chi-square or Fisher exact test, as appropriate. Benjamini–Hochberg false discovery rate correction [40] was applied separately to the eight omnibus Kruskal–Wallis tests and to the eight prespecified active-versus-inactive pairwise comparisons. Multivariable logistic regression models were considered secondary analyses and are reported with nominal two-sided *p* values without an additional multiplicity correction. Adipokine comparisons in Behçet’s disease are known to be sensitive to demographic adjustment [10]. Each analyte was therefore entered on a base-2 logarithmic scale into a separate binary logistic regression model for active-versus-inactive disease, together with age, sex, body mass index and disease duration. Odds ratios accordingly express the change in odds of active disease per doubling of concentration, and the model therefore contained the biomarker together with four covariates, which was as much as the sample size allowed. Discriminative performance was quantified by the area under the receiver operating characteristic curve, with cut-off values derived from the Youden index. Confidence intervals for areas under the curve, and for sensitivity and specificity at the Youden threshold, were obtained by non-parametric bootstrapping with 5000 resamples. Correlated curves were compared using the DeLong method [41], with the difference in area under the curve reported alongside its confidence interval and *p* value. Associations with clinical and laboratory variables were assessed with Spearman’s rank correlation. Membership of the index followed a single prespecified rule. An analyte entered the index if it differed between active and inactive disease after Benjamini–Hochberg correction (q < 0.05), joining the positive arm if its median was higher in active disease and the negative arm if it was lower. Six of the eight analytes met this criterion; CTRP9 and CTRP13 did not and were left out. Internal validity was then examined by bootstrapping with 1000 resamples. Both the composition of the index and its scaling were derived from these data. The entire procedure was therefore repeated inside each bootstrap sample: the Benjamini–Hochberg selection, the assignment of each analyte to an arm, the z-scaling and the calculation of the index. The resulting index was then evaluated in the observations left out of that sample. Of the 1000 resamples, 860 yielded a non-empty arm on both sides and contributed to this estimate. A composite index was constructed by averaging the z-transformed base-2 logarithms of the analytes that rose in active disease and subtracting the corresponding average of those that fell. Z scores were computed from the mean and standard deviation of the log-transformed values among the 60 patients with Behçet’s disease. The standardisation therefore used all 60 patients, whereas every performance and inferential estimate was obtained in the 58 with complete covariate data. All performance and inferential analyses involving the composite index were carried out in those same 58 patients. These comprise the receiver operating characteristic curves for the index, for C-reactive protein and for the two combined, the Youden threshold and the classification counts derived from it, the DeLong comparisons and the bootstrap validation. Restricting them in this way keeps adjusted and unadjusted estimates within one sample, and it lets the correlated curves compared by the DeLong method rest on identical observations. Membership of the two arms was therefore defined by the observed direction of change in this cohort rather than by an a priori functional classification, which makes the index a data-derived construct. Its incremental value over C-reactive protein was assessed by entering both into a single logistic model. Descriptive statistics, group comparisons, and logistic regression analyses were performed using IBM SPSS Statistics, version 26.0 (IBM Corp., Armonk, NY, USA). The Benjamini–Hochberg adjustment, bootstrap confidence intervals, DeLong’s test for comparing correlated receiver operating characteristic curves, and internal validation of the composite index were implemented in Python version 3.12.3 (Python Software Foundation, Wilmington, DE, USA) using NumPy 2.4.4, SciPy 1.17.1, statsmodels 0.15.0 and scikit-learn 1.8.0. All resampling procedures used a fixed random seed, so that every estimate reported here derives from a single analysis run. A false discovery rate-adjusted q value < 0.05 was considered statistically significant. For secondary analyses, two-sided *p* values < 0.05 were considered nominally significant.

## 3. Results

### 3.1. Participants

The three groups were comparable in age (median 42.0, 41.5 and 41.0 years for controls, inactive and active disease; *p* = 0.755) and sex distribution (*p* = 0.277). Body mass index differed modestly across groups (24.0, 23.0 and 22.0 kg/m^2^; *p* = 0.012) and was retained as a covariate throughout. C-reactive protein was higher in active disease (median 6.5 mg/L) than in inactive disease or controls (2.0 mg/L in both; *p* < 0.001). The erythrocyte sedimentation rate did not differ significantly (*p* = 0.486). The activity score separated the two patient groups as expected (7.0 vs. 1.0; *p* < 0.001). Disease duration was similar in active and inactive disease (123 vs. 108 months; *p* = 0.499), so differences between the patient groups cannot be attributed to disease chronicity. Colchicine was used by all but two patients (29 in each group). Conventional immunosuppressants were more frequent in active disease (18 vs. 10 patients; azathioprine 18 vs. 10, methotrexate 1 vs. 0, cyclophosphamide 2 vs. 0, mycophenolate mofetil 1 vs. 0). Overall use of tumour necrosis factor inhibitors was similar (16 vs. 15), with a shift from infliximab (10 vs. 15) towards adalimumab (8 vs. 2). Twenty-one patients had been exposed to systemic corticosteroids at some point in the disease course, distributed almost equally between the two groups (10 with inactive and 11 with active disease; *p* = 1.000). Treatment was recorded as cumulative exposure rather than as therapy in use on the day of sampling, so these counts describe what each patient had received over the course of the disease. No healthy control was taking immunomodulatory medication. Biomarker concentrations did not differ according to prior exposure to a tumour necrosis factor inhibitor (all *p* ≥ 0.12; composite index *p* = 0.68) or to corticosteroids (all *p* ≥ 0.08; composite index *p* = 0.52). Among the conventional immunosuppressants, only CTRP12 showed a borderline difference (*p* = 0.048), with no effect on the composite index (*p* = 0.11). C-reactive protein was likewise unrelated to either treatment class. Full characteristics are given in Table 1.

### 3.2. Biomarker Concentrations Across Groups

**Figure 1:**
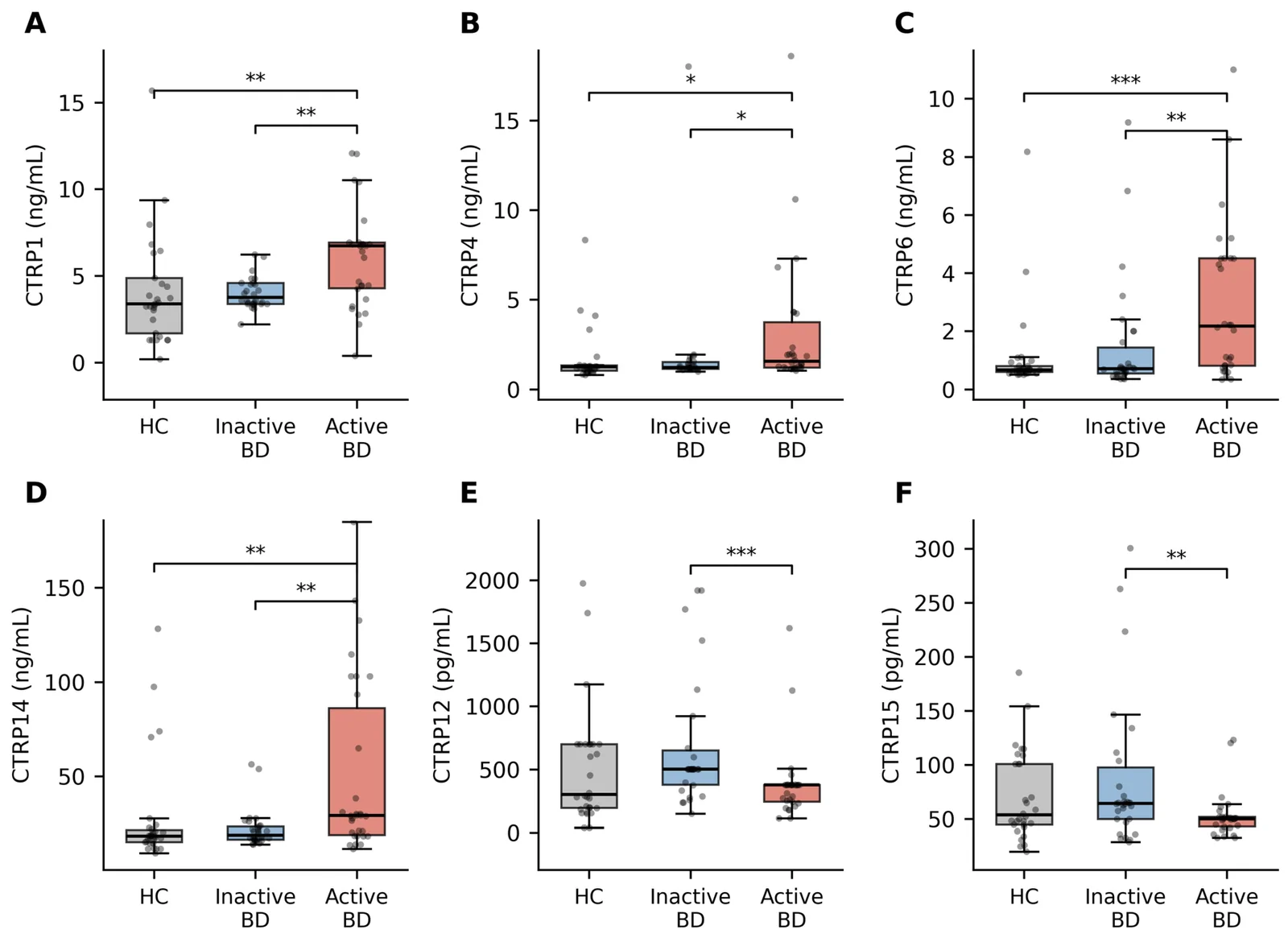
Serum concentrations of the six analytes that differed between active and inactive disease after correction for multiple testing. Analytes higher in active disease are shown in panels (**A**–**D**), whereas analytes lower in active disease are shown in panels (**E**,**F**). Boxes show the median and interquartile range; whiskers extend to 1.5 × IQR, and individual observations are overlaid as dots, each representing one participant. Significance annotations denote unadjusted pairwise Mann–Whitney U *p* values (* *p* < 0.05, ** *p* < 0.01, *** *p* < 0.001); the false discovery rate-adjusted values are given in Table 2; Table 3. HC, healthy controls; BD, Behçet’s disease.

Five of the eight analytes differed significantly across the three groups and retained significance after correction of the omnibus comparison (CTRP6, CTRP14, CTRP1, CTRP12 and CTRP4), and CTRP15 fell just short of the corrected threshold (q = 0.062). Inspection of the pairwise comparisons showed that most of this variation arose from the contrast between active and inactive disease rather than between patients and controls, and CTRP9 and CTRP13 did not differ between any groups. Not every change was monotonic across the three groups. CTRP12, for example, was highest in inactive disease (501 pg/mL), intermediate in active disease (376 pg/mL) and lowest in controls (302 pg/mL). Neither patient–control contrast therefore reached significance, despite a clear difference between the two patient groups (Table 2, Figure 1).

### 3.3. A Bidirectional Pattern Differs Between Active and Inactive Disease

All six analytes differed between active and inactive disease after false discovery rate correction. Four analytes were higher in active disease: CTRP6 increased from 0.71 to 2.17 ng/mL (q = 0.009), CTRP14 from 18.9 to 29.3 ng/mL (q = 0.007), CTRP1 from 3.75 to 6.73 ng/mL (q = 0.015) and CTRP4 from 1.22 to 1.56 ng/mL (q = 0.041). Two analytes were lower: CTRP12 decreased from 501 to 376 pg/mL (q = 0.004) and CTRP15 from 64.5 to 50.3 pg/mL (q = 0.015). Four associations were nominally significant in logistic models adjusted for age, sex, body mass index and disease duration. The odds of active disease per doubling of concentration were 0.27 for CTRP12 (95% confidence interval 0.11–0.71; *p* = 0.008), 2.73 for CTRP14 (1.16–6.44; *p* = 0.022), 1.74 for CTRP6 (1.13–2.69; *p* = 0.012) and 0.37 for CTRP15 (0.14–0.93; *p* = 0.035). CTRP1 (odds ratio 1.79, 0.88–3.65; *p* = 0.107) and CTRP4 (1.45, 0.88–2.39; *p* = 0.141) did not retain nominal significance after adjustment. The evidence for these two analytes therefore rests on the false discovery rate-adjusted pairwise comparison alone, not on an independently significant multivariable association. For the members whose immunological actions are better established, the observed directions were broadly compatible with the reported functional literature; CTRP4 and CTRP14, however, lack sufficient functional characterisation for such classification. Among the six analytes that differed between active and inactive disease, single-analyte areas under the curve ranged from 0.663 (95% confidence interval 0.516–0.799) for CTRP4 to 0.762 (0.631–0.882) for CTRP12 (Table 3).

### 3.4. Correlation with Clinical Activity

The analytes that differed between the patient groups also correlated with the Behçet’s Disease Current Activity Form score in the directions predicted by the group comparisons: CTRP14 (rho = 0.38), CTRP15 (rho = −0.38), CTRP12 (rho = −0.32), CTRP6 (rho = 0.30) and CTRP1 (rho = 0.28), all *p* < 0.05. Correlations with C-reactive protein were weaker and less consistent, and no analyte correlated with disease duration. The only associations with demographic variables were weak inverse correlations of CTRP12 with age (rho = −0.28) and of CTRP14 with body mass index (rho = −0.27). This supports the retention of these variables as covariates (Table 4).

### 3.5. Performance of a Composite Index

**Figure 2:**
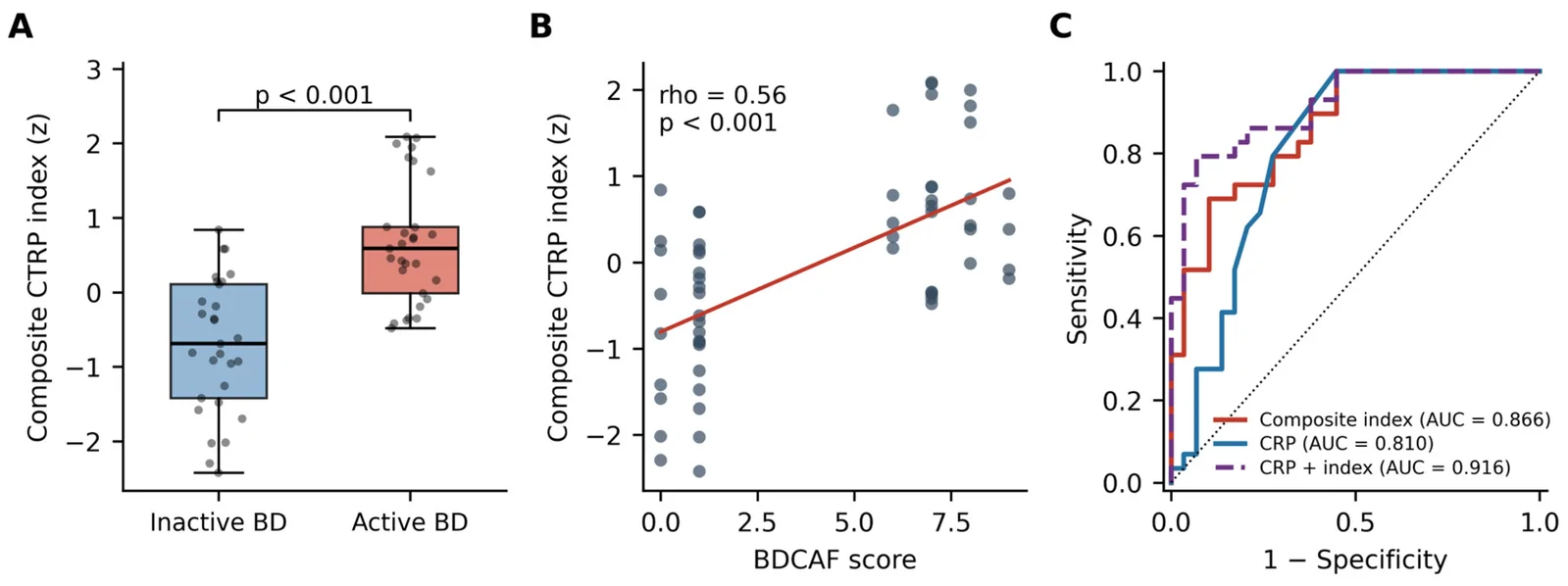
Composite CTRP index. (**A**) Index values in inactive and active disease, with each dot representing one patient. (**B**) Correlation between the index and the Behçet’s Disease Current Activity Form score; each dot is one patient and the red line is a least-squares linear fit shown to indicate the direction of the association, the reported coefficient being Spearman’s rho. (**C**) Receiver operating characteristic curves for the index, C-reactive protein and the two combined.

Because the members moved in opposite directions, a composite index was constructed as the mean z-score of CTRP1, CTRP4, CTRP6 and CTRP14 minus the mean z-score of CTRP12 and CTRP15. The index was higher in active than in inactive disease (*p* < 0.001) and achieved an area under the curve of 0.866 (95% confidence interval 0.765–0.944). The adjusted odds ratio was 9.1 per standard deviation (2.6–31.5; *p* < 0.001). At the Youden threshold of 0.30, twenty of the twenty-nine patients with active disease and twenty-six of the twenty-nine with inactive disease were classified correctly, giving a sensitivity of 20/29 (69%; 95% confidence interval 52–85) and a specificity of 26/29 (90%; 77–100). The denominator is twenty-nine rather than thirty because all analyses of the composite index were confined to the 58 patients with complete covariate data, so that adjusted and unadjusted estimates refer to an identical sample. Disease duration was missing for two patients; the index itself could have been calculated for all sixty, but restricting every estimate to the same 58 patients avoids comparing figures drawn from different subsets. The index correlated with the activity score at rho = 0.56 (*p* < 0.001). C-reactive protein alone achieved an area under the curve of 0.810 (0.682–0.915) and the two combined 0.916 (0.839–0.974). Formal comparison of the correlated curves by the DeLong method showed that adding the index to C-reactive protein produced a small increment whose confidence interval included zero (difference in area under the curve +0.106, 95% confidence interval −0.002 to +0.214; *p* = 0.055); an increment this close to the boundary should be regarded as provisional rather than established. Neither the comparison of the index with C-reactive protein alone (+0.056, −0.100 to +0.212; *p* = 0.482) nor that of the combined model with the index alone (+0.050, −0.017 to +0.117; *p* = 0.142) reached significance. The index nevertheless remained associated with active disease in a model containing C-reactive protein (*p* = 0.002), suggesting that it captures information not fully represented by C-reactive protein within this dataset. Because the index was both derived and evaluated in these data, its performance was examined using bootstrapping. The whole derivation was repeated inside each bootstrap sample, so that the selection of the components, their assignment to an arm and the z-scaling were all validated rather than the scaling alone. Across 1000 resamples the out-of-bag area under the curve was 0.812 (95% confidence interval 0.649–0.946); 860 resamples yielded a non-empty arm on both sides and contributed to this estimate. This last figure is the appropriate estimate of what might be expected in new data, and the gap from the apparent value indicates that about 0.05 of the discrimination reported here reflects the index having been defined in the same cohort. These analyses do not demonstrate superior discrimination over C-reactive protein (Table 5, Figure 2).

## 4. Discussion

This is the first time the C1q/TNF-related protein family has been examined in Behçet’s disease. Active disease was accompanied by a pattern that moved in two directions at once: CTRP1, CTRP4, CTRP6 and CTRP14 were higher; CTRP12 and CTRP15 were lower. Six analytes differed between active and inactive disease after correction for multiple testing. Four of them held up after adjustment for age, sex, body mass index and disease duration, and five of the six correlated with the clinical activity score in the same directions. A composite index built from these opposing changes was higher in active disease, with an area under the curve of 0.866, and it stayed associated with activity after adjustment for C-reactive protein. Its incremental discrimination over C-reactive protein alone did not reach statistical significance.

The internal consistency of the pattern is notable, but the functional interpretation should be made cautiously. The evidence is strongest for CTRP1 and CTRP6. Adipose CTRP1 is induced by tumour necrosis factor alpha and interleukin-1 beta [17]. It couples macrophage lipid handling to vascular inflammation [18] and worsens cardiac fibrosis and post-infarction inflammation [19,20]. CTRP6 links adipose inflammation to insulin resistance [23], amplifies the macrophage inflammatory response [24] and suppresses anti-inflammatory, M2-like polarisation [25]. CTRP12 moved in the opposite direction and has repeatedly shown protective actions, including limitation of inflammasome activation [30], promotion of M2-like polarisation [31] and inverse associations with coronary disease burden and inflammatory markers [27,28,29]. CTRP15 also decreased in active disease and has been grouped with protective CTRPs in experimental frameworks [9], although clinical data are not uniform and higher circulating concentrations have been reported in coronary artery disease [32]. CTRP4 and CTRP14 cannot presently be assigned confidently to a pro-inflammatory functional category; their placement in the increasing arm of the composite reflects the observed direction in this cohort rather than independent mechanistic evidence. For several members, then, the bidirectional pattern is biologically plausible. Whether these proteins drive the inflammatory process or simply mirror it is beyond what a cross-sectional study can settle.

The non-monotonic behaviour of CTRP12 deserves comment, since it does not fit a simple model in which a protective member is depleted by disease. Concentrations were highest in inactive disease and lowest in controls. The fall that comes with a flare therefore starts from a level above that of healthy individuals. One reading is compensatory. Quiescent disease may be accompanied by a rise in protective members, which is then lost once inflammation becomes clinically apparent. On this view CTRP12 reports on state rather than on disease itself, which would fit both its being the analyte most strongly associated with activity and its showing no patient–control difference at all. This reading is speculative, and at least four alternatives fit the data equally well. Treatment is one. Patients in remission were on therapy, and while tumour necrosis factor inhibitor use showed no association with any analyte, conventional immunosuppressant use was borderline for CTRP12 itself. A drug effect sitting on top of the disease effect remains possible. Disease stage is another, since a protein that rises with cumulative disease exposure would produce the same ordering without any compensatory mechanism. Metabolic differences may also contribute, as CTRP12 is an adipose-derived protein and body mass index differed modestly across the groups. Finally, the possibility of analytical variability should be acknowledged, since a single non-duplicated measurement per participant provides no internal estimate of measurement error. Longitudinal sampling of the same patients through flare and remission would distinguish between these explanations. Circulating adipokine findings in Behçet’s disease are themselves heterogeneous: leptin and resistin have been reported as elevated [10,11], whereas visfatin and omentin have been reported as reduced [10,12,13]. This heterogeneity cautions against mapping serum concentration directly onto a simple pro-inflammatory or anti-inflammatory label. Our data point the same way: the relative pattern across several members looks more informative than any one concentration taken alone.

From a practical standpoint, the composite index is the most clinically relevant exploratory result, but its relationship to C-reactive protein requires careful interpretation. C-reactive protein is a hepatic acute-phase reactant synthesised in response to interleukin-6, whereas CTRPs are adipose-derived mediators with immunometabolic actions and are not acute-phase proteins. In the present dataset, adding the index to C-reactive protein increased the area under the curve by 0.106, but the confidence interval included zero and the comparison did not reach conventional significance (*p* = 0.055), so the increment is unproven. Conversely, the index remained associated with active disease when C-reactive protein was included in the same model (*p* = 0.002), suggesting that the CTRP pattern is not simply a second measurement of the acute-phase response. This distinction may be relevant in Behçet’s disease, in which acute-phase reactants do not consistently parallel clinical manifestations; in our cohort, for example, the erythrocyte sedimentation rate did not differ between active and inactive disease. The index was, however, derived and evaluated in the same sample, so its apparent performance is optimistic and requires independent validation. Clinical translation would also be challenging: C-reactive protein is measured rapidly and inexpensively on routine automated analysers, whereas the present CTRP measurements required separate research-grade enzyme-linked immunosorbent assays with several hours of processing and no established clinical laboratory pathway. Any future clinical score would therefore need to be reduced to a small, reproducible analyte set on a multiplex or automated platform and then shown prospectively to improve decision-making. The present data do not support the replacement of, or superiority over, C-reactive protein.

Treatment needs separate consideration. Every patient had been treated, and the two groups had not been exposed to the same agents. Conventional immunosuppressants were more common in active disease, whereas exposure to a tumour necrosis factor inhibitor was similar and only the choice of agent differed. What we can examine is cumulative exposure rather than therapy in use at the moment of sampling, which limits what any treatment analysis here can settle: a drug taken years earlier and one taken that week are counted alike. Two directions of bias are conceivable. Effective suppression of inflammation in the remission group would lower the pro-inflammatory members and raise the protective ones, giving the observed gradient with no independent disease effect at all. Or the drugs may act on adipose-derived proteins directly, in which case the gradient is pharmacology rather than disease activity. These analyses gave no evidence of a large treatment-related effect. Corticosteroid use was almost identical in the two groups (10 vs. 11 patients; *p* = 1.000), was unrelated to any analyte and to the composite index, and adding it as a covariate left every estimate essentially unchanged: the adjusted odds ratio for the composite index moved from 9.1 to 9.6 (95% confidence interval 2.7–34.3; *p* < 0.001), and the four individually significant analytes retained their significance. Beyond corticosteroids, neither analytes nor the composite index differed according to prior exposure to a tumour necrosis factor inhibitor, and among the conventional immunosuppressants only CTRP12 showed a borderline difference. C-reactive protein was likewise unrelated to either treatment class, so the acute-phase comparison is not distorted by differential therapy. These analyses are underpowered for subgroup comparison, treatment was not randomised, and doses and duration were not systematically recorded. Drug exposure is also inseparable from disease state in an observational design, so residual confounding by therapy remains possible. Two observations merit separate comment. CTRP14 rose in active disease despite an almost complete absence of prior immunological characterisation [33,34,35]; without a mechanistic framework, this finding should be regarded as hypothesis-generating. The sexually dimorphic expression reported experimentally [33] was reflected in a borderline sex difference in our cohort, which is why sex was retained in all adjusted models. Second, CTRP6 rose with activity even though it has been shown to act as an endogenous complement regulator whose administration ameliorates induced arthritis [26]. This apparent tension may reflect a counter-regulatory rise in response to inflammation rather than a causal contribution, or divergence between tissue and circulating compartments of the kind documented for CTRP1 in chronic kidney disease [21]. Both CTRP4 and CTRP12 have been reported as elevated in Hashimoto’s thyroiditis [42]. That matches our direction for CTRP4 but runs opposite to what we found for CTRP12. Organ-specific autoimmunity and systemic vasculitis may simply behave differently here. Distinguishing these possibilities will require longitudinal sampling through treated flares.

Several limitations apply. The design is cross-sectional and cannot establish whether CTRP changes precede, accompany or follow clinical activity; sampling the same patients in flare and remission is the necessary next step and would also allow within-patient responsiveness to be assessed. With ninety participants, subgroup analysis was limited: vascular and neurological involvement were too infrequent for meaningful examination. No a priori power calculation was performed, and with eight analytes, three-group comparisons, regression models and receiver operating characteristic analyses, the study may be underpowered for some of the secondary questions, so the effect estimates should be read as provisional. Recruitment was quota-based and stratified by clinically judged activity, with enrolment stopped at thirty patients per group, so the study population is not a consecutive series. Patients with intermediate activity were not recruited into either stratum, which is why no BDCAF score between 2 and 5 appears in the data. Comparing the two ends of the activity range sharpens the contrast and is likely to have inflated the effect estimates relative to a full clinical spectrum; how the analytes or the composite index behave in patients with intermediate activity, which is where a marker would be most useful, cannot be judged from these data. Every patient had been treated, and many had received immunosuppressive or biologic therapy, so an effect of treatment on circulating concentrations remains possible. Treatment was recorded as cumulative exposure rather than as therapy in use on the day of sampling, and neither doses nor duration were captured, so exposure could be modelled only as ever or never. A drug influencing these proteins while it is being taken would be poorly detected by such a variable, and the treatment analyses reported here should be read with that in mind. BD is managed with continuous therapy once diagnosed and an untreated comparison group could not ethically be assembled; the concentrations reported here are those of a treated population and may not represent the profile of untreated disease. Systemic corticosteroid and TNF inhibitor use was similar between the two patient groups, whereas conventional immunosuppressants were numerically more frequent in active disease. Treatment-class analyses showed no clear association with the composite index, although CTRP12 showed a borderline association with conventional immunosuppressant use. Adjustment for corticosteroid exposure did not materially alter the estimates, arguing against a dominant treatment effect; nevertheless, residual confounding by therapy cannot be excluded. Doses and duration were not systematically recorded, however, so exposure could be modelled only as present or absent and a dose-dependent influence on adipose-derived proteins cannot be ruled out, although disease duration itself did not differ between the patient groups. A study in newly diagnosed, treatment-naive patients, or a longitudinal design sampling the same patients before and after treatment is started, would separate the contribution of disease activity from that of therapy more cleanly than is possible here.

The composite index is data-derived, and we built and tested it in the same cohort with no held-out validation set. Bootstrap internal validation, repeating the selection of components in each resample, gave an out-of-bag area under the curve of 0.812 against an apparent 0.866, so the optimism is real but modest. The bootstrap confidence intervals around the apparent value nevertheless take no account of the uncertainty introduced by choosing the components from these same data. The composition of the index is data-derived in a second sense: the two arms were defined by the direction each analyte took in this cohort, so grouping CTRP4 and CTRP14 with the pro-inflammatory members is provisional and needs independent functional and clinical support. Samples were measured once rather than in duplicate, so no within-study estimate of analytical imprecision is available, although all analytes were assayed on a single plate with a single reagent lot and the operator was blinded to group. Randomisation of sample order was not documented. Activity was defined by a clinical instrument rather than by an objective standard, which is unavoidable in this disease but introduces classification uncertainty, although the bimodal distribution of scores meant that no patient lay close to the threshold. We measured the analytes for which commercial assays were available, not a set chosen on biological grounds, so the panel does not cover the family systematically and untested members may behave differently. The labels we use, pro-inflammatory and protective, come from experimental work in mice and largely from metabolic and cardiovascular models; we have assumed that they carry over to human vasculitis, and that assumption is untested. Serum tells us nothing about the tissues in which these proteins act, and for CTRP1 at least the two compartments are known to move in opposite directions. All patients came from one tertiary centre in a population where the disease is common, so neither the absolute concentrations nor the associations need hold elsewhere, and patients whose disease required urgent escalation of treatment were not systematically enrolled, so the active group reflects routine outpatient practice rather than the full range of severity. The kit-specific assay ranges and analytical sensitivities given in Section 2.2 were taken from the manufacturer’s inserts and were not independently verified in our laboratory. The kits are also not widely validated in the published literature, and absolute concentrations should not be set against studies using other platforms; confirmation with assays from a second manufacturer, and inclusion of adiponectin as a reference analyte, would give more confidence in the measurements.

## 5. Conclusions

In this cross-sectional study, active Behçet’s disease was associated with a coordinated bidirectional pattern across the C1q/TNF-related protein family, with CTRP1, CTRP4, CTRP6 and CTRP14 higher and CTRP12 and CTRP15 lower than in inactive disease. A composite index derived from these opposing changes differed markedly between the two groups and remained associated with activity after adjustment for C-reactive protein. However, the improvement in discrimination obtained by adding the index to C-reactive protein did not reach conventional statistical significance, and the index was derived and evaluated in the same cohort. These findings therefore identify an exploratory CTRP pattern associated with active disease rather than a validated biomarker. Longitudinal confirmation in the same patients through flare and remission, external validation in an independent cohort, and analytical validation using duplicate measurements on a second assay platform would all be required before the score could be considered for monitoring or clinical decision-making.

## Figures and Tables

**Table 1:** Demographic, clinical and laboratory characteristics of the study population.

Variable	Healthy Controls (*n* = 30)	Inactive BD (*n* = 30)	Active BD (*n* = 30)	*p*
Age, years	42.00 (36.50–43.75)	41.50 (33.25–45.75)	41.00 (34.00–47.50)	0.755
Female/male, *n*	13/17	9/21	15/15	0.277
Body mass index, kg/m^2^	24.00 (22.00–25.00)	23.00 (22.00–23.00)	22.00 (21.00–23.00)	0.012
C-reactive protein, mg/L	2.00 (1.25–3.00)	2.00 (1.00–3.75)	6.50 (4.00–17.00)	<0.001
Erythrocyte sedimentation rate, mm/h	14.00 (7.25–19.75)	11.00 (7.25–21.50)	16.50 (9.00–24.75)	0.486
White blood cells, ×10^9^/L	7.52 (6.47–8.69)	7.41 (5.98–10.03)	7.07 (6.15–9.45)	0.978
Neutrophil-to-lymphocyte ratio	1.65 (1.31–2.15)	2.01 (1.37–2.78)	1.75 (1.36–2.60)	0.256
Platelets, ×10^9^/L	310.50 (261.00–339.25)	261.00 (234.50–284.50)	254.50 (225.00–292.75)	0.003
Albumin, g/dL	4.80 (4.70–4.97)	4.63 (4.50–4.90)	4.60 (4.50–4.70)	0.002
Disease duration, months	—	108.00 (48.00–154.00)	123.00 (98.00–149.00)	0.499
BDCAF score	—	1.00 (0.00–1.00)	7.00 (7.00–8.00)	<0.001
Mucocutaneous involvement, n	0	30	30	—
Ever ocular involvement, *n*	0	14	11	—
Ever articular involvement, *n*	0	4	1	—
Ever vascular involvement, *n*	0	5	4	—
Ever neurological involvement, *n*	0	0	4	—
Systemic corticosteroid, *n*	0	10	11	1.000
Colchicine, *n*	0	29	29	1.000
Azathioprine, *n*	0	10	18	0.069
Other conventional immunosuppressant, *n*	0	0	4	—
Any conventional immunosuppressant, *n*	0	10	18	0.069
Infliximab, *n*	0	15	10	0.295
Adalimumab, *n*	0	2	8	0.080
Etanercept, *n*	0	0	1	1.000
Any TNF inhibitor, *n*	0	15	16	1.000

Data are median (interquartile range) unless stated otherwise. Treatment rows record agents received over the course of the disease rather than concurrent therapy, and the categories are not mutually exclusive: most patients received more than one agent, and five received more than one TNF inhibitor, so the agent-specific counts sum to more than the number of patients in the “Any TNF inhibitor” row. Continuous variables were compared across the three groups with the Kruskal–Wallis test; disease duration and BDCAF score were compared between the two patient groups with the Mann–Whitney U test and sex with the chi-square test. Individual treatments were compared between the two patient groups with the Fisher exact test, and the resulting *p* values are given in the *p* column; no treatment comparison reached the conventional threshold. Counts of cumulative organ involvement are descriptive and were not formally compared. BD, Behçet’s disease; BDCAF, Behçet’s Disease Current Activity Form; TNF, tumour necrosis factor.

**Table 2:** Serum CTRP concentrations across study groups.

Biomarker (Unit)	Healthy Controls	Inactive BD	Active BD	KW *p*	KW q	HC vs. In	HC vs. Act	In vs. Act
CTRP1 (ng/mL)	3.38 (1.70–4.88)	3.75 (3.38–4.59)	6.73 (4.29–6.92)	0.004	0.011	0.143	0.005	0.009
CTRP4 (ng/mL)	1.25 (1.05–1.34)	1.22 (1.14–1.52)	1.56 (1.21–3.76)	0.023	0.037	0.355	0.016	0.031
CTRP6 (ng/mL)	0.67 (0.60–0.81)	0.71 (0.55–1.44)	2.17 (0.81–4.51)	0.001	0.005	0.888	<0.001	0.003
CTRP9 (ng/mL)	8.40 (5.35–15.25)	7.55 (7.12–9.04)	7.76 (6.11–14.72)	0.988	0.988	0.888	0.970	0.842
CTRP12 (pg/mL)	301.54 (194.76–700.00)	501.07 (379.00–651.04)	375.85 (244.35–376.82)	0.011	0.022	0.178	0.594	<0.001
CTRP13 (pg/mL)	607.75 (268.22–772.41)	510.50 (507.25–597.28)	554.37 (431.22–694.70)	0.307	0.351	0.160	0.496	0.301
CTRP14 (ng/mL)	18.48 (15.35–21.79)	18.91 (16.70–23.56)	29.25 (19.11–86.25)	0.001	0.005	0.482	0.002	0.002
CTRP15 (pg/mL)	53.75 (45.00–101.00)	64.50 (50.00–97.91)	50.25 (43.25–52.00)	0.047	0.062	0.336	0.264	0.008

Data are median (interquartile range). KW, Kruskal–Wallis; q, Benjamini–Hochberg false discovery rate-adjusted *p* value across the eight analytes; HC, healthy controls; In, inactive; Act, active.

**Table 3:** Association of each analyte with active disease in patients with Behçet’s disease.

Biomarker (Unit)	Inactive BD	Active BD	*p*	q	Adjusted OR	95% CI	Adjusted *p*	AUC	Cut-Off	Sens., %	Spec., %
CTRP12 (pg/mL)	501.07 (379.00–651.04)	375.85 (244.35–376.82)	<0.001	0.004	0.27	0.11–0.71	0.008	0.762 (0.631–0.882)	≤380.00	87	73
CTRP14 (ng/mL)	18.91 (16.70–23.56)	29.25 (19.11–86.25)	0.002	0.007	2.73	1.16–6.44	0.022	0.735 (0.596–0.865)	≥29.00	57	93
CTRP6 (ng/mL)	0.71 (0.55–1.44)	2.17 (0.81–4.51)	0.003	0.009	1.74	1.13–2.69	0.012	0.721 (0.586–0.848)	≥0.81	77	70
CTRP15 (pg/mL)	64.50 (50.00–97.91)	50.25 (43.25–52.00)	0.008	0.015	0.37	0.14–0.93	0.035	0.699 (0.557–0.837)	≤52.00	80	70
CTRP1 (ng/mL)	3.75 (3.38–4.59)	6.73 (4.29–6.92)	0.009	0.015	1.79	0.88–3.65	0.107	0.696 (0.545–0.843)	≥6.42	57	93
CTRP4 (ng/mL)	1.22 (1.14–1.52)	1.56 (1.21–3.76)	0.031	0.041	1.45	0.88–2.39	0.141	0.663 (0.516–0.799)	≥1.86	47	83
CTRP13 (pg/mL)	510.50 (507.25–597.28)	554.37 (431.22–694.70)	0.301	0.344	0.53	0.19–1.44	0.214	0.578 (0.424–0.733)	≥524.86	73	63
CTRP9 (ng/mL)	7.55 (7.12–9.04)	7.76 (6.11–14.72)	0.842	0.842	1.05	0.64–1.72	0.848	0.516 (0.364–0.671)	≤7.30	50	73

Models are adjusted for age, sex, body mass index and disease duration and are based on the 58 patients with complete covariate data. Odds ratios express the change in odds of active disease per doubling of concentration. The *p* and q columns refer to the unadjusted active-versus-inactive Mann–Whitney U comparison and its Benjamini–Hochberg correction across the eight analytes, respectively; adjusted-model *p* values are nominal and were not subjected to a second multiplicity correction. Cut-off values were derived from the Youden index; areas under the curve are given with bootstrap 95% confidence intervals (5000 resamples). OR, odds ratio; CI, confidence interval; AUC, area under the receiver operating characteristic curve.

**Table 4:** Spearman correlations between analytes and clinical variables in patients.

Biomarker	BDCAF	CRP	ESR	NLR	Disease Duration	Age	BMI
CTRP1	+0.28 *	+0.08	−0.10	−0.10	+0.01	−0.09	−0.02
CTRP4	+0.21	+0.21	−0.06	−0.06	−0.04	−0.11	−0.04
CTRP6	+0.30 *	+0.29 *	+0.07	−0.03	+0.02	−0.05	+0.05
CTRP9	+0.02	−0.01	−0.13	−0.02	−0.15	+0.06	−0.05
CTRP12	−0.32 *	−0.31 *	−0.06	+0.08	+0.13	−0.28 *	+0.21
CTRP13	+0.05	+0.16	+0.12	+0.16	−0.02	+0.00	−0.16
CTRP14	+0.38 *	+0.16	−0.01	−0.14	+0.09	+0.05	−0.27 *
CTRP15	−0.38 *	−0.25	−0.10	+0.21	−0.03	−0.23	+0.21

* *p* < 0.05. BDCAF, Behçet’s Disease Current Activity Form; CRP, C-reactive protein; ESR, erythrocyte sedimentation rate; NLR, neutrophil-to-lymphocyte ratio; BMI, body mass index.

**Table 5:** Performance of the composite CTRP index for active disease.

Metric	Value
*p* value, composite index, active vs. inactive disease	<0.001
Area under the curve, composite index (95% CI)	0.866 (0.765–0.944)
Sensitivity at Youden threshold (0.30), *n/N* (95% CI)	20/29, 69% (52–85)
Specificity at Youden threshold (0.30), *n/N* (95% CI)	26/29, 90% (77–100)
Out-of-bag AUC, component selection repeated in each resample (95% CI)	0.812 (0.649–0.946)
Adjusted odds ratio per 1 SD (95% CI)	9.1 (2.6–31.5)
*p* value, adjusted model	<0.001
Correlation with BDCAF score (rho)	0.56
Area under the curve, C-reactive protein alone (95% CI)	0.810 (0.682–0.915)
Area under the curve, C-reactive protein + composite index (95% CI)	0.916 (0.839–0.974)
*p* value for composite index in C-reactive protein-adjusted model	0.002
ΔAUC, C-reactive protein + index vs. C-reactive protein alone (DeLong)	+0.106 (−0.002 to +0.214), *p* = 0.055

The composite index was defined as the mean z-transformed base-2 logarithm of the concentrations of CTRP1, CTRP4, CTRP6 and CTRP14 minus that of CTRP12 and CTRP15. The adjusted model contains age, sex, body mass index and disease duration. All performance and inferential analyses of the composite index—the index, C-reactive protein, the combined model, the DeLong comparisons and the bootstrap validation—were performed in the same 58 patients (29 active, 29 inactive) with complete covariate data, so that adjusted and unadjusted estimates refer to an identical sample. CI, confidence interval.

## Data Availability

The data presented in this study are available upon request from the corresponding author.
